# Synthesis and Antitumor Activity of Amino Acid Ester Derivatives Containing 5-Fluorouracil

**DOI:** 10.3390/molecules14093142

**Published:** 2009-08-25

**Authors:** Jing Xiong, Hai-Feng Zhu, Ya-Juan Zhao, Yun-Jun Lan, Ji-Wang Jiang, Jing-Jing Yang, Shu-Feng Zhang

**Affiliations:** 1 College of Chemistry and Materials Engineering, Wenzhou University, Wenzhou 325000, China; 2 State Key Laboratory of Fine Chemicals, Dalian University of Technology, Dalian 116012, China

**Keywords:** 5-fluorouracil, amino acid ester, antitumor activity

## Abstract

A series of amino acid ester derivatives containing 5-fluorouracil were synthesized using 1-ethyl-3-(3-dimethylaminopropyl)carbodiimide hydrochloride (EDC·HCl) and *N*-hydroxybenzotriazole (HOBt) as a coupling agent. The structures of the products were assigned by NMR, MS, IR etc. The *in vitro* antitumor activity tests against leukaemia HL-60 and liver cancer BEL-7402 indicated that (*R*)-ethyl 2-(2-(5-fluoro-2,4-dioxo-3,4-dihydropyrimidin-1(2*H*)-yl)acetamido)-3-(4-hydroxyphenyl) propanoate showed more inhibitory effect against BEL-7402 than 5-FU.

## 1. Introduction

5-Fluorouracil (5-FU) is an antimetabolite of the pyrimidine analogue type, which is frequently used for treating solid tumors, such as colorectal, gastric tract, and liver carcinomas [1,2,3]. However, the clinical applications of 5-FU are greatly limited by its short plasma half-life, poor tumor affinity, myelosuppression, and strong intestinal toxicity. Consequently, numerous research efforts have focused on the discovery of suitable carrier-linked prodrugs, in which 5-FU is conjugated with a wide spectrum of low- or high- molecular-weight carriers including glucose, peptides, and biodegradable polymers such as polysaccharides, liposomes, etc [4,5,6,7,8,9,10]. In general prodrug systems the drug is bound to the carrier through a spacer that incorporates a predetermined breaking point that allows the bound drug to be released at the cellular target site. Therefore, the optimization physicochemical properties of a carrier, the modification of the carrier with 5-FU to preserve the targeting properties of the carrier and ensure a controlled release of 5-FU inside or outside the tumor cells are the critical aspects of 5-FU prodrug design [3,11].

Peptides play an important role in human metabolism. Some peptide derivatives of 5-FU have been reported as an approach to develop chemotherapeutic agents with improved physicochemical and biological characteristics [4,12,13], and we also have previously reported some peptide derivatives of 5-FU [14,15,16]. In continuation of the research, we now describe our studies on the synthesis and assessment of some amino acid ester derivatives containing 5-FU with the aim of finding appropriate biodegradable linkages.

## 2. Results and Discussion

### 2.1. Chemistry

**Scheme 1:**
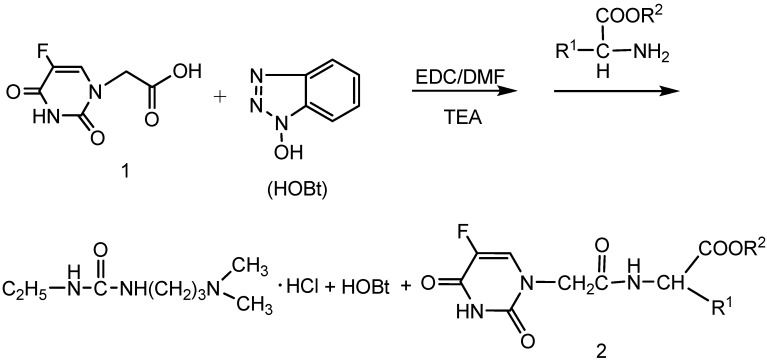
Synthesis of 2-(5-fluoro-2,4-dioxo-3,4-dihydropyrimidin-1(2*H*)-yl)-aceto amino acid ester derivatives **2**.

**Figure 1:**
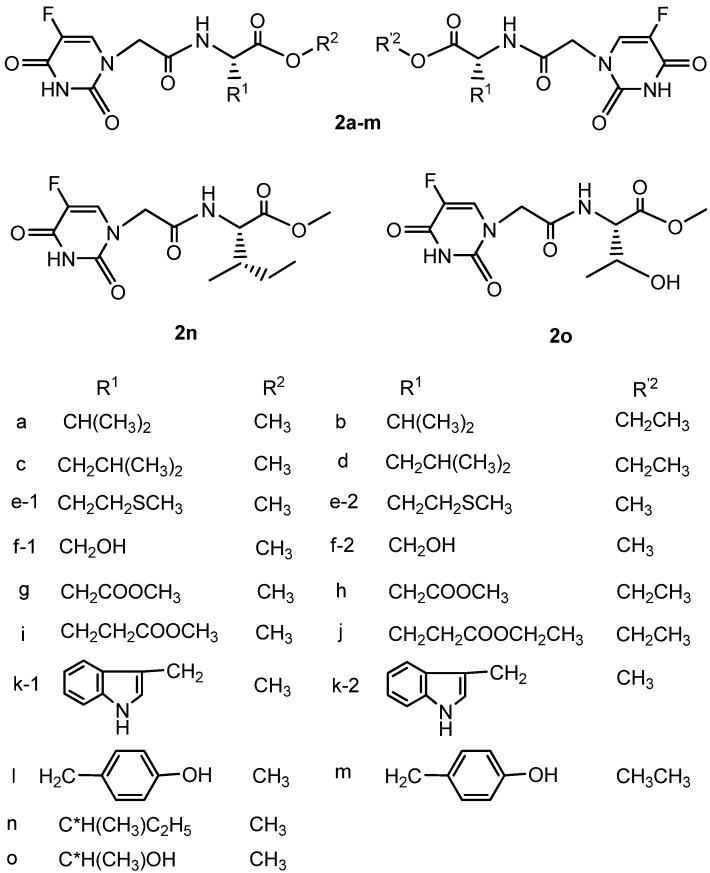
Structural formulae of compounds **2**.

The synthetic route to the target compounds **2a-o** is shown in Scheme 1 and Figure 1. The starting material 2-(5-fluoro-2,4-dioxo-3,4-dihydropyrimidin-1(2*H*)-yl) acetic acid (or 5-fluorouracil-1-yl acetic acid) (**1**) could be easily prepared by carboxymethylation of 5-fluorouracil according to the literature [17]. Treatment of compound **1** with a series of amino acid esters using 1-ethyl-3-(3-dimethylamino-propyl)carbodiimide hydrochloride (EDC·HCl) and *N*-hydroxybenzotriazole (HOBt) as a coupling agent yielded a series of 2-(5-fluoro-2,4-dioxo-3,4-dihydropyrimidin-1(2*H*)-yl)-aceto amino acid ester derivatives **2**. HOBt was reported as a racemisation suppressant in peptide coupling reactions with carbodiimide coupling reagents [18,19,20].

The purity and structures of compounds **2a-o** were established on the basis of their melting points, specific rotations and spectral data, which were in full agreement with the proposed molecular structures. The ^1^H-NMR spectra of all compounds showed doublets at 7.93-8.02 ppm, which corresponded to the coupling of fluorine and hydrogen signals in the FC=CH moieties. Compounds **2e-1** and **2e-2**, for example, almost have the same melting point (139-140 °C), the same spectral data, but opposite specfic rotations of ${\left[\alpha \right]}_{D}^{18.1}$−10.4 and ${\left[\alpha \right]}_{D}^{18.1}$+10.4, respectively. In the ^1^H-NMR their CH_2_SCH_3_ fragment methylene protons were observed as multiplets at δ 2.50-2.41 ppm, which overlapped with the signal of the solvent DMSO-*d*_6_. The ^13^C-NMR of **2c** and **2d** displayed signals at δ 39.7 ppm from the methylene carbon from the CH_2_CH(CH_3_)_2_ moiety which overlapped as well with that of the solvent DMSO-*d*_6_,. The assignment of the above four compounds were further proven by ^13^C-^1^H COSY spectra.

### 2.2. In vitro antitumor activity

All target compounds **2a-o** were evaluated for their *in vitro* antitumor activity against the HL-60 leukaemia and BEL-7402 liver cancer cell lines by the 3-(4,5-dimethylthiazol-2-yl)-2,5-diphenyl-2H-tetrazoliumbromide (MTT) [21] and Sulforhodamine B (SRB) assay methods [22], respectively, with 5-FU and the prodrug FT-207 being used for comparisons (Table 1 and Table 2).

As shown in Table 1, all compounds’ *in vitro* inhibition rates against HL-60 were significantly lower than that of 5-FU, except for the R-type compounds **2h**, **2j**, **2k-2** and **2m**, which exhibited equivalent inhibitory effect as 5-FU at 10^-4^ mol/L concentration, but the activity decreased rapidly when the concentration declined. The results indicate that these compounds were less sensitive to HL-60 at lower concentrations when the *N*-1 position of 5-FU was occupied.

In Table 2, almost all the compounds showed less sensitivity to BEL-7402, except **2m**, which showed more potent inhibitory effect than 5-FU. The reason maybe was the *R*-conformation of **2m** with a moderately rigid stereo structure, being composed of the pyrimidine ring and the phenyl ring, so it could release 5-FU sufficiently, while other compounds showed either more flexible configurations (such as **2a-e**), or a more rigid structure such as the case of **2k** [23]. The different inhibition against HL-60 and BEL-7402 between *R*-type and *S*-type compounds suggested the complexity of the antitumor mechanism.

## 3. Experimental

### 3.1. General

Melting points of synthesized compounds were determined on a Digital Melting Point Appatatus X-4 and were uncorrected. Mass spectra were obtained on a DECAX-30000 LCQ DecaXP Plus instrument. IR spectra were recorded (in KBr) on a Bruker EQUINOX 55. ^1^H-NMR and ^13^C-NMR were recorded on Bruker AVANCE-300 at 300 and 75 MHz, respectively in DMSO-*d*_6_ solutions with TMS as internal standard.

### 3.2. General procedure for the synthesis of compounds ***2a-o***

Synthesis of compounds **2a-o** was accomplished as shown in Scheme 1. 2-(5-Fluoro-2,4-dioxo-3,4-dihydropyrimidin-1(2*H*)-yl) acetic acid (10 mmol), HOBt (10 mmol) and DMF (50 mL) were added to a round-bottom flask, then EDC·HCl (13 mmol), L- or D-amino acid ester hydrochloride (10 mmol), and triethylamine (20 mmol) were added to the above mixture. After 10 h reaction at room temperature with thin layer chromatography (TLC) monitoring, the white solid 5-fluorouracil-1-yl-aceto amino acid esters **2a-o** were obtained after filtration, reduced pressure distillation of DMF, and silica gel column chromatography separation.

*(S)-Methyl 2-(2-(5-fluoro-2,4-dioxo-3,4-dihydropyrimidin-1(2H)-yl)acetamido)-3-methylbutanoate* (**2a**). Yield: 68%; m.p. 109-110°C; ^1^H-NMR *δ*: 11.80 (s, 1H, NH of 5-FU), 8.54 (d, 1H, NH, *J* = 8.1 Hz), 8.01 (d, 1H, FC=CH, ^3^*J*_FH_ = 6.9 Hz), 4.39 (d, 2H, NCH_2_, *J* = 16.8 Hz), 4.22 (t, 1H, NCH, *J* = 7.2 Hz), 3.65 (s, 3H, OCH_3_), 2.09-1.98 (m, 1H, CCH, *J* = 6.6 Hz), 0.89 (d, 3H, CH_3_, *J* = 6.6 Hz), 0.87 (d, 3H, CH_3_, J = 6.6 Hz); ^13^C-NMR *δ*: 171.9, 167.1, 157.7(d, ^2^*J*_FC_ = 25.6 Hz), 149.8, 139.3 (d, ^1^*J*_FC_ = 226.7 Hz), 131.3 (d, ^2^*J*_FC_ = 33.6 Hz), 57.7, 51.9, 49.5, 30.4, 19.0, 18.3; IR (cm^-1^) *ν*: 3456, 3280, 2969, 1722, 1666, 1560, 1467, 1379, 1227, 1146, 783; MS (ESI) *m/z*: 300 (M^-^); ${\left[\alpha \right]}_{D}^{10.0}$ -22.0 (*c* 1.0, DMF).

(*R*)-*Ethyl 2-(2-(5-fluoro-2,4-dioxo-3,4-dihydropyrimidin-1(2H)-yl)acetamido)-3-methylbutanoate* (**2b**). Yield: 65%; m.p. 136-137 °C; ^1^H-NMR *δ*: 11.83(s, 1H, NH of 5-FU), 8.53 (d, 1H, NH, *J* = 8.1 Hz), 8.02 (d, 1H, FC=CH, ^3^*J*_FH_ = 6.6 Hz), 4.38 (d, 2H, NCH_2_, *J* = 16.8 Hz), 4.19 (dd, 1H, NCH, *J* = 8.1, 6.3 Hz), 4.16-4.05 (m, 2H, COOCH_2_, *J* = 7.2 Hz), 2.11-1.98 (m, 1H, CCH), 1.19 (t, 3H, OCH_2_CH_3_, *J* = 7.2 Hz), 0.89 (d, 3H, CH_3_, *J* = 6.9 Hz), 0.88 (d, 3H, CH_3_, *J* = 6.6 Hz); ^13^C-NMR *δ*: 174.0, 169.6, 160.5 (d, ^2^*J*_FC_ = 25.3 Hz), 151.6, 141.2 (d, ^1^*J*_FC_ = 228.1 Hz), 132.8 (d, ^2^*J*_FC_ = 33.5 Hz), 63.3, 59.9, 51.6; 31.6, 20.0, 19.2, 15.2; IR (cm^-1^) *ν*: 3295, 3253, 2977, 1707, 1552, 1467, 1377, 1225, 1148; MS (ESI) *m*/*z*: 314(M^-^); ${\left[\alpha \right]}_{D}^{18.0}$+14.0 (*c* 0.1, DMF).

(*S*)-*Methyl 2-(2-5-(fluoro-2,4-dioxo-3,4-dihydropyrimidin-1(2H)-yl)acetamido)-4-methylpentanoate* (**2c**). Yield: 75%; m.p. 144-145°C; ^1^H-NMR *δ*: 11.83 (d, 1H, NH of 5-FU, ^4^*J*_FH_ = 5.4 Hz), 8.60 (d, 1H, NH, *J* = 7.8 Hz), 8.02 (d, 1H, FC=CH, ^3^*J*_FH_ = 6.9 Hz), 4.32 (s, 2H, NCH_2_), 4.35-4.27 (m, 1H, NCH), 3.62 (s, 3H, OCH_3_), 1.66-1.59 (m, 1H, CCH), 1.54-1.46 (m, 2H, CCH_2_), 0.88 (d, 3H, CH_3_, *J* = 6.3 Hz), 0.83 (d, 3H, CH_3_, *J* = 6.3 Hz); ^13^C-NMR *δ* 172.9, 167.0, 157.8 (d, ^2^*J*_FC_ = 25.5 Hz), 149.8, 139.4 (d, ^1^*J*_FC_ = 226.6 Hz), 131.3 (d, ^2^*J*_FC_ = 33.8 Hz), 52.2, 50.5, 49.6, 39.7, 24.3, 22.9, 21.6; IR (cm^-1^) *ν*: 3323, 3046, 2959, 1666, 1543, 1472, 1384, 1244, 1155; MS (ESI) *m/z*: 314(M^-^); ${\left[\alpha \right]}_{D}^{16.1}$-19.2 (*c* 1.0, DMF).

(*R*)-*Ethyl 2-(2-5-(fluoro-2,4-dioxo-3,4-dihydropyrimidin-1(2H)-yl)acetamido)-4-methylpentanoate* (**2d**). Yield: 72%; m.p. 146-147°C; ^1^H-NMR *δ*: 11.81(d, 1H, NH of 5-FU, ^4^*J*_FH_ = 5.1 Hz), 8.57(d, 1H, NH, *J* =7.8 Hz), 8.01(d, 1H, FC=CH, ^3^*J*_FH_ = 6.9 Hz), 4.32(s, 2H, NCH_2_), 4.30-4.24(m, 1H, NCH), 4.08(q, 2H, OCH_2_, *J* = 7.2 Hz), 1.67-1.58(m, 1H, CCH), 1.56-1.48(m, 2H, CCH_2_), 1.17(t, 3H, OCH_2_CH_3_, *J* = 7.2 Hz), 0.89(d, 3H, CH_3_, *J* = 6.3 Hz), 0.84(d, 3H, CH_3_, *J* = 6.3 Hz); ^13C^-NMR(75 MHz) *δ*: 172.3, 166.8, 157.7(d, ^2^*J*_FC_ = 25.2 Hz), 149.7, 139.3(d, ^1^*J*_FC_ = 226.4 Hz), 131.2 (d, ^2^*J*_FC_ = 33.9 Hz), 60.7, 50.6, 49.5, 39.7, 24.3, 22.8, 21.5, 14.1; IR(KBr, cm^-1^) *ν*: 3328, 2963, 2818, 1690, 1637, 1555, 1473, 1377, 1238, 1196, 1150; MS(ESI) *m/z*: 328(M^-^); ${\left[\alpha \right]}_{D}^{17.7}$+11.6(*c* 1.0, DMF).

(*S*)-methyl 2-(2-5-(fluoro-2,4-dioxo-3,4-dihydropyrimidin-1(2*H*)-yl)acetamido)-4-(methylthio)butanoate (**2e-1**). Yield: 62%; m.p. 139-140°C; ^1^H-NMR(300 MHz) *δ*: 11.84(s, 1H, NH of 5-FU), 8.64(d, 1H, NH, *J* = 7.5 Hz), 8.02(d, 1H, FC=CH, ^3^*J* = 6.9 Hz), 4.46-4.39(m, 1H, NCH), 4.33(s, 2H, NCH_2_), 3.63(s, 3H, OCH_3_), 2.50-2.48(m, 2H, CH_2_S), 2.02(s,3H, SCH_3_), 1.97-1.79(m, 2H, CCH_2_); ^13^C-NMR(75 MHz) *δ*: 172.2, 167.1, 157.8(d, ^2^*J*_FC_ = 25.7 Hz), 149.9, 139.5(d, ^1^*J*_FC_ = 226.5 Hz), 131.2(d, ^2^*J*_FC_ = 33.8 Hz), 52.3, 51.1, 49.7, 30.9, 29.5, 14.8; IR(KBr, cm^-1^) *ν*: 3345, 3042, 2983, 1687, 1662, 1542, 1474, 1428, 1386, 1233; MS(ESI) *m/z*: 332(M^-^); ${\left[\alpha \right]}_{D}^{18.1}$-10.4(*c* 1.0, DMF).

(*R*)-methyl 2-(2-5-(fluoro-2,4-dioxo-3,4-dihydropyrimidin-1(2*H*)-yl)acetamido)-4-(methylthio)butanoate (**2e-2**). Yield: 59%; m.p. 138-140 °C; ^1^H-NMR(300 MHz) *δ*: 11.83(s, 1H, NH of 5-FU), 8.63(d, NH, *J* = 7.6 Hz), 8.02(d, 1H, FC=CH, ^3^*J*_FH_ = 6.0 Hz), 4.43-4.38(m, 1H, NCH), 4.33(s, 2H, NCH_2_), 3.64(s, 3H, OCH_3_), 2.47-2.43(m, 2H, CH_2_S), 2.03(s, 3H, SCH_3_), 1.95-1.87(m, 2H, CCH_2_); ^13C-NMR^(75 MHz) *δ*: 172.1, 167.1, 157.8(d, ^2^*J*_FC_ = 25.7 Hz), 149.8, 139.5(d, ^1^*J*_FC_ = 226.7 Hz), 131.2 (d, ^2^*J*_FC_ = 33.7 Hz), 52.3, 51.1, 49.6, 30.9, 29.5, 14.7; IR(KBr, cm^-1^) *ν*: 3348, 3237, 2962, 1725, 1660, 1569, 1478, 1425, 1381, 1345, 1305, 1246, 1162, 794; MS(ESI) *m/z*: 332(M^-^); ${\left[\alpha \right]}_{D}^{18.1}$+10.4(*c* 1.0, DMF).

(*S*)-methyl 2-(2-5-(fluoro-2,4-dioxo-3,4-dihydropyrimidin-1(2*H*)-yl)acetamido)-3-hydroxypropanoate (**2f-1**). Yield: 54%; m.p. 190-191°C; ^1^H-NMR(300 MHz) *δ*: 11.83(d, 1H, NH of 5-FU, ^4^*J*_HH_ = 4.5 Hz), 8.63(d, 1H, NH, *J* = 8.1 Hz), 8.01(d, 1H, FC=CH, ^3^*J*_FH_ = 6.9 Hz), 5.11(t, 1H, OH, *J* = 5.4 Hz), 4.38(s, 2H, NCH_2_), 4.42-4.36(m, 1H, NCH), 3.65(s, 3H, OCH_3_), 3.75-3.58(m, 2H, CH_2_OH, *J* = 5.4, 10.8 Hz); ^13C-NMR^(75 MHz) *δ*: 170.9, 167.0, 157.6(d, ^2^*J*_FC_ = 25.7 Hz), 149.8, 139.3(d, ^1^*J*_FC_ = 226.7 Hz), 131.2(d, ^2^*J*_FC_ = 33.7 Hz), 61.4, 54.8, 52.0, 49.4; IR(KBr, cm^-1^) *ν*: 3459, 3295, 2850, 1713, 1692, 1563, 1467, 1416, 1382, 1277, 1251, 1185, 1071; MS(ESI) *m*/*z*: 288(M^-^). ${\left[\alpha \right]}_{D}^{10.7}$-10.0 (*c* 1.0, DMF).

*(R)-methyl 2-(2-5-(fluoro-2,4-dioxo-3,4-dihydropyrimidin-1(2H)-yl)acetamido)-3-hydroxypropanoate* (**2f-2**). Yield: 55%; m.p. 190-192°C; ^1^H-NMR(300 MHz) *δ*: 11.81(s, 1H, NH of 5-FU), 8.62(d, 1H, NH, *J* = 7.5 Hz), 8.00(d, 1H, FC=CH, ^3^*J*_FH_ = 6.9 Hz), 5.12(t, 1H, OH, *J* = 5.4 Hz), 4.37(s, 2H, NCH_2_), 4.41-4.35(m, 1H, NCH), 3.63(s, 3H, OCH_3_), 3.74-3.56(m, 2H, CH_2_OH, *J* = 5.4, 10.8 Hz); ^13C-NMR^(75 MHz) *δ*: 171.0, 167.0, 157.7(d, ^2^*J*_FC_ = 25.6 Hz), 149.8, 139.3(d, ^1^*J*_FC_ = 226.6 Hz), 131.4(d, ^2^*J*_FC_ = 33.5 Hz), 61.5, 54.9, 52.1, 49.5; IR(KBr, cm^-1^) *ν*: 3459, 3295, 2850, 1713, 1692, 1563, 1467, 1416, 1382, 1277, 1251, 1185, 1071; MS(ESI) *m/z*: 288(M^-^). ${\left[\alpha \right]}_{D}^{11.0}$+10.0 (*c* 1.0, DMF).

(*S*)-dimethyl 2-(2-5-(fluoro-2,4-dioxo-3,4-dihydropyrimidin-1(2*H*)-yl)acetamido)succinate (**2g**). Yield: 65%; m.p. 145-147 °C; ^1^H-NMR(300 MHz) *δ*: 11.85(d, 1H, NH of 5-FU, ^4^*J*_FH_ = 5.1 Hz), 8.76(d, 1H, NH, *J* =7.8 Hz), 8.01(d, 1H, FC=CH, ^3^*J*_FH_ = 6.6 Hz), 4.68(dd, 1H, NCH, *J* =7.5, 6.6 Hz), 4.33(s, 2H, NCH_2_), 3.64(s, 3H, OCH_3_), 3.62(s, 3H, OCH_3_), 2.85-2.70(m, 2H, CCH_2_, *J* = 6.6, 7.5, 16.8 Hz); ^13C-NMR^(75 MHz) *δ*: 170.9, 170.5, 166.9, 157.7(d, ^2^*J*_FC_ = 25.7 Hz), 149.8, 139.4(d, ^1^*J*_FC_ = 228.0 Hz), 131.1 (d, ^2^*J*_FC_ = 33.9 Hz), 52.4, 51.9, 49.5, 48.7, 35.9; IR(KBr, cm^-1^) *ν*: 3348, 3191, 2850, 1755, 1697, 1526, 1430, 1380, 1337, 1216, 1047, 980; MS(ESI) *m/z*: 330(M^-^); ${\left[\alpha \right]}_{D}^{11.0}$-14.4(*c* 1.0, DMF).

(*R*)-diethyl 2-(2-5-(fluoro-2,4-dioxo-3,4-dihydropyrimidin-1(2*H*)-yl)acetamido)succinate (**2h**). Yield: 68%; m.p. 116-118 °C; ^1^H-NMR(300 MHz) *δ*: 11.82(d, 1H, NH of 5-FU, ^4^*J*_FH_ = 4.8 Hz), 8.71(d, 1H, NH, *J* = 7.8 Hz), 7.99(d, 1H, FC=CH, ^3^*J*_FH_ = 6.8 Hz), 4.63(dd, 1H, NCH, *J* = 6.6, 7.5 Hz), 4.33(s, 2H, NCH_2_), 4.12-4.03(m, 4H, OCH_2_, *J* = 6.9 Hz), 2.81-2.65(m, 2H, CCH_2_, *J* = 6.6, 16.5 Hz), 1.17(t, 3H, CH_3_, *J* = 6.9 Hz), 1.16(t, 3H, CH_3_, *J* = 6.9 Hz); ^13C-NMR^(75 MHz) *δ*: 170.4, 170.0, 166.9, 157.7(d, ^2^*J*_FC_ = 25.7 Hz), 149.8, 139.4(d, ^1^*J*_FC_ = 228.3 Hz), 131.2 (d, ^2^*J*_FC_ = 33.8 Hz), 61.2, 60.6, 49.5, 48.9, 36.1, 14.2, 14.1; IR(KBr, cm^-1^) *ν*: 3314, 3217, 2990, 1721, 1691, 1549, 1467, 1378, 1339, 1240, 1167, 1021, 792; MS(ESI) *m/z*: 358(M^-^); ${\left[\alpha \right]}_{D}^{9.7}$+16.0 (*c* 1.0, DMF).

(*S*)-dimethyl 2-(2-5-(fluoro-2,4-dioxo-3,4-dihydropyrimidin-1(2*H*)-yl)acetamido)pentanedioate (**2i**). Yield: 42%; m.p. 147-148 °C; ^1^H-NMR(300 MHz) *δ*: 11.85(s, 1H, NH of 5-FU), 8.63(d, 1H, NH, *J* =7.8 Hz), 8.02(d, 1H, FC=CH, ^3^*J*_FH_ = 6.9 Hz), 4.32(s, 2H, NCH_2_), 4.37-4.34(m, 1H, NCH), 3.63(s, 3H, OCH_3_), 3.58(s, 3H, OCH_3_), 2.37(t, 2H, CH_2_CH_2_CO, *J* = 7.5 Hz), 2.06-1.76(m, 2H, CH_2_CH_2_CO); ^13C-NMR(75 MHz)^ *δ*: 172.7, 171.9, 168.0, 157.7(d, ^2^*J*_FC_ = 25.7 Hz), 149.8, 139.4(d, ^1^*J*_FC_ = 226.7 Hz), 131.1(d, ^2^*J*_FC_ = 33.7 Hz), 52.2, 51.5, 51.3, 49.6, 29.6, 26.4; IR(KBr, cm^-1^) *ν*: 3328, 2964, 1716, 1660, 1541, 1449, 1348, 1261, 800; MS(ESI) *m/z*: 344(M^-^); ${\left[\alpha \right]}_{D}^{17.8}$-8.39 (*c* 0.5, DMF).

(*R*)-diethyl 2-(2-5-(fluoro-2,4-dioxo-3,4-dihydropyrimidin-1(2*H*)-yl)acetamido)pentanedioate (**2j**). Yield: 45%; m.p. 108-109 °C; ^1^H-NMR(300 MHz) *δ*: 11.84(s, 1H, NH of 5-FU), 8.61(d, 1H, NH, *J* = 7.8 Hz), 8.01(d, 1H, FC=CH, ^3^*J*_FH_ = 6.9 Hz), 4.33(s, 2H, NCH_2_), 4.30-4.26(m, 1H, NCH), 4.08(q, 2H, OCH_2_CH_3_, *J* = 6.9 Hz), 4.04(q, 2H, OCH_2_CH_3_, *J* = 7.2 Hz), 2.36(t, 2H, CH_2_CH_2_CO, *J* = 7.5 Hz), 2.03-1.78(m, 2H, CH_2_CH_2_CO), 1.17(t, 3H, OCH_2_CH_3_, *J* = 6.9 Hz), 1.16(t, 3H, OCH_2_CH_3_, *J* = 7.2 Hz); ^13C-NMR(75 MHz)^ *δ*: 172.2, 171.4, 167.0, 157.7(d, ^2^*J*_FC_ = 25.7 Hz), 149.8, 139.4(d, ^1^*J*_FC_ = 226.9 Hz), 131.2(d, ^2^*J*_FC_ = 33.7 Hz), 60.9, 60.1, 51.5, 49.6, 29.9, 26.5, 14.2, 14.1; IR(KBr, cm^-1^) *ν*: 3304, 3213, 2924, 1725, 1675, 1546, 1468, 1416, 1379, 1250, 1176, 1023; MS(ESI) *m/z*: 372 (M^-^); ${\left[\alpha \right]}_{D}^{8.7}$+12.40 (*c* 1.0, DMF).

(*S*)-methyl 2-(2-5-(fluoro-2,4-dioxo-3,4-dihydropyrimidin-1(2*H*)-yl)acetamido)-3-(1*H*-indol-3-yl)pro-panoate (**2k-1**). Yield: 50%; m.p. 205-206 °C; ^1^H-NMR(300 MHz) *δ*: 11.84(s, 1H, NH of 5-FU), 10.90(s, 1H, NH of indole), 8.72(d, 1H, NH, *J* = 7.2 Hz), 7.93(d, 1H, FC=CH, ^3^*J*_FH_ = 6.8 Hz), 7.48(d, 1H, Ar-H, *J* = 7.5 Hz), 7.33(d, 1H, Ar-H, *J* = 8.1 Hz), 7.16(d, 1H, =CHN, *J* = 2.2 Hz), 7.07(t, 1H, Ar-H, *J* = 8.1, 6.9 Hz), 6.99(t, 1H, Ar-H, *J* = 7.5, 6.9 Hz), 4.54(dd, 1H, NCH, *J* = 7.2, 6.6 Hz), 4.33(d, 2H, NCH_2_, *J* = 16.5 Hz), 3.56(s, 3H, OCH_3_), 3.19-3.03(m, 2H, CCH_2_, *J* = 7.5, 6.0, 14.4 Hz); ^13C-NMR^(75 MHz) *δ*: 172.2, 166.9, 157.8(d, ^2^*J*_FC_ = 25.7 Hz), 149.9, 139.4(d, ^1^*J*_FC_ = 228.1 Hz), 136.3, 131.3(d, ^2^*J*_FC_ = 33.8 Hz), 127.3, 124.1, 121.2, 118.7, 118.2, 111.7, 109.2, 53.6, 52.1, 49.5, 27.4; IR(KBr, cm^-1^) *ν*: 3386, 3312, 3069, 2977, 1702, 1545, 1381, 1233, 1062; MS(ESI) *m/z*: 387(M^-^); ${\left[\alpha \right]}_{D}^{17.9}$+36.4 (*c* 1.0, DMF).

(*R*)-methyl 2-(2-5-(fluoro-2,4-dioxo-3,4-dihydropyrimidin-1(2*H*)-yl)acetamido)-3-(1*H*-indol-3-yl)pro-panoate (**2k-2**). Yield: 53%; m.p. 205-206 °C; ^1^H-NMR(300 MHz) *δ*: 11.84(d, 1H, NH of 5-FU, ^4^*J*_FH_ = 5.1 Hz), 10.90(s, 1H, NH of indole), 8.72(d, 1H, NH, *J* = 7.5 Hz), 7.93(d, 1H, FC=CH, ^3^*J*_FH_ = 6.9 Hz), 7.48(d, 1H, Ar-H, *J* = 7.8 Hz), 7.34(d,1H, Ar-H, *J* = 8.1 Hz), 7.16(s, 1H, =CHN), 7.07(t, 1H, Ar-H, *J* = 7.2, 7.8 Hz), 6.99(t, 1H, Ar-H, *J* = 7.2 Hz), 4.55(dd, 1H, NCH, *J* = 6.9, 6.6 Hz), 4.33(d, 2H, NCH_2_, *J* = 16.5 Hz), 3.57(s, 3H, OCH_3_), 3.20-3.03(m, 2H, CCH_2_, *J* = 7.5, 6.0, 14.8 Hz); ^13C-NMR^(75 MHz) *δ*: 172.2, 166.9, 157.8 (d, ^2^*J*_FC_ = 25.7 Hz), 149.9, 139.4(d, ^1^*J*_FC_ = 228.2 Hz), 136.3, 131.3(d, ^2^*J*_FC_ = 33.8 Hz), 127.3, 124.1, 121.2, 118.7, 118.2, 111.7, 109.2, 53.5, 52.1, 49.5, 27.4; IR(KBr, cm^-1^) *ν*: 3392, 3320, 3075, 1732, 1648, 1542, 1446, 1385, 1355, 1248, 1219,1099, 744; MS(ESI) *m/z*: 387(M^-^); ${\left[\alpha \right]}_{D}^{17.8}$-36.4 (*c* 1.0, DMF).

(*S*)-methyl 2-(2-5-(fluoro-2,4-dioxo-3,4-dihydropyrimidin-1(2*H*)-yl)acetamido)-3-(4-hydroxyphenyl) propanoate (**2l**). Yield: 72%; m.p. 192-193 °C; ^1^H-NMR(300 MHz) *δ*: 11.85(s, 1H, NH of 5-FU), 9.28(s, 1H, OH), 8.68(d, 1H, NH, *J* = 7.5 Hz), 7.94(d, 1H, FC=CH, ^3^*J*_FH_ = 6.9 Hz), 6.99 (d, 2H, Ar-H, *J* = 8.4 Hz), 6.66 (d, 2H, Ar-H, *J* = 8.4 Hz), 4.39(dd, 1H, NCH, *J* = 6.3, 7.5 Hz), 4.31(d, 2H, NCH_2_, *J* = 16.8 Hz), 3.59(s, 3H, OCH_3_), 2.93-2.77(m, 2H, CH_2_Ar, *J* = 6.3, 8.1, 13.8 Hz); ^13^C-NMR(75 MHz) *δ*: 171.9, 166.9, 157.7(d, ^2^*J*_FC_ = 25.7 Hz), 156.3, 149.8, 139.4(d, ^1^*J*_FC_ = 226.9 Hz), 131.2(d, ^2^*J*_FC_ = 33.8 Hz), 130.3, 127.0, 115.3, 54.4, 52.0, 49.5, 36.3; IR(KBr, cm^-1^) *ν*: 3271, 1739, 1712, 1661, 1552, 1516, 1451, 1386, 1231, 1164, 778; MS(ESI) *m/z*: 364(M^+^); ${\left[\alpha \right]}_{D}^{17.8}$+16.6 (*c* 1.0, DMF).

(*R*)-ethyl 2-(2-5-(fluoro-2,4-dioxo-3,4-dihydropyrimidin-1(2*H*)-yl)acetamido)-3-(4-hydroxyphenyl) propanoate (**2m**). Yield: 70%; m.p. 156-158 °C; ^1^H-NMR(300 MHz) *δ*: 11.82(s, 1H, NH of 5-FU), 9.23(s, 1H, OH), 8.63(d, 1H, NH, *J* = 7.5 Hz), 7.94(d, 1H, FC=CH, ^3^*J*_FH_ = 6.6 Hz), 6.99(d, 2H, Ar-H, *J* = 8.4 Hz), 6.65(d, 2H, Ar-H, *J* = 8.4 Hz), 4.36(dd, 1H, NCH, *J* = 7.5, 6.9 Hz), 4.31(d, 2H, NCH_2_, *J* = 16.8 Hz), 4.02(q, 2H, OCH_2_, *J* = 7.2 Hz), 2.89-2.77(m, 2H, CH_2_Ar, *J* = 7.5, 6.0, 13.8 Hz), 1.10(t, 3H, CH_3_, *J* = 7.2 Hz); ^13C-NMR^(75 MHz) *δ*: 171.4, 166.9, 157.7(d, ^2^*J*_FC_ = 25.7 Hz), 156.3, 149.8, 139.4(d, ^1^*J*_FC_ = 228.1 Hz), 131.2(d, ^2^*J*_FC_ = 33.9 Hz), 130.3, 127.0, 115.3, 60.7, 54.4, 49.5, 36.4, 14.1; IR(KBr, cm^-1^) *ν*: 3336, 3065, 2851, 1691, 1532, 1380, 1340, 1220, 801; MS(ESI) *m/z*: 378(M^-^); ${\left[\alpha \right]}_{D}^{9.7}$-24.4 (*c* 1.0, DMF).

(2*S,*3*S*)-methyl 2-(2-5-(fluoro-2,4-dioxo-3,4-dihydropyrimidin-1(2*H*)-yl)acetamido)-3-methylpentanoate (**2n**). Yield: 70%; m.p. 152-154 °C; ^1^H-NMR(300 MHz) *δ*: 11.82(d, 1H, NH of 5-FU, ^4^*J*_FH_ = 5.1 Hz), 8.57(d, 1H, NH, *J* = 8.1 Hz), 8.01(d, 1H, FC=CH, ^3^*J*_FH_ = 6.9 Hz), 4.36(d, 2H, NCH_2_, *J* = 16.8 Hz), 4.25(dd, 1H, NCH, *J* = 8.1, 6.6 Hz), 3.63(s, 3H, OCH_3_), 1.77-1.72(m, 1H, CCH), 1.43-1.09(m, 2H, CCH_2_), 0.84(t, 3H, CH_2_CH_3_, *J* = 7.2 Hz), 0.83(d, 3H, CHCH_3_, *J* = 6.9 Hz); ^13C-NMR^(75 MHz) *δ*: 172.0, 167.1, 157.7(d, ^2^*J*_FC_ = 25.7 Hz), 149.8, 139.3(d, ^1^*J*_FC_ = 227.9 Hz), 131.4(d, ^2^*J*_FC_ = 33.8 Hz), 56.6, 52.0, 49.5, 36.9, 24.9, 15.6, 11.3; IR(KBr, cm^-1^) *ν*: 3275, 3083, 2968, 1712, 1666, 1571, 1460, 1378, 1340, 1243, 1149, 976, 700; MS(ESI) *m/z*: 314(M^-^); ${\left[\alpha \right]}_{D}^{17.5}$-3.0 (*c* 0.5, DMF).

(2*S,*3*S*)-methyl 2-(2-5-(fluoro-2,4-dioxo-3,4-dihydropyrimidin-1(2*H*)-yl)acetamido)-3-hydroxybutanoate (**2o**). Yield: 47%; m.p. 207-208 °C; ^1^H-NMR(300 MHz) *δ*: 11.79(d, 1H, NH of 5-FU, ^4^*J*_FH_ = 5.1 Hz), 8.44(d, 1H, NH, *J* = 8.4 Hz), 8.01(d, 1H, FC=CH, ^3^*J*_FH_ = 6.6 Hz), 5.03(d, 1H, OH, *J* = 5.1 Hz), 4.42(dd, 2H, NCH_2_, *J* = 6.6, 16.5 Hz), 4.32(dd, 1H, NCH, *J* = 8.4, 3.3 Hz), 4.15-4.08(m, 1H, CHOH, *J* = 3.3, 5.1, 6.3 Hz), 1.05(d, 3H, CH_3_, *J* = 6.3 Hz); ^13^C NMR(75 MHz) *δ* 171.0, 167.4, 157.7(d, ^2^*J*_FC_ = 25.6 Hz), 149.8, 139.3(d, ^1^*J*_FC_ = 226.6 Hz), 131.4(d, ^2^*J*_FC_ = 33.5 Hz), 66.5, 58.1, 52.0, 49.6, 20.2; IR(KBr, cm^-1^) *ν*: 3484, 3312, 2977, 1718, 1663, 1558, 1376, 1283, 1238, 1140; MS(ESI) *m/z*: 302(M^-^); ${\left[\alpha \right]}_{D}^{17.6}$-2.0 (*c* 0.1, DMF).

## 4. Conclusions

A serials of amino acid ester derivatives containing 5-fluorouracil were synthesized by EDC/HOBt method and characterized. The *in vitro* antitumor activity tests indicated that the synthesized compounds had less inhibition rates against HL-60 and BEL-7402 than 5-FU except compound **2m**, which showed more potent inhibitory effect against BEL-7402 than 5-FU. This might be explained by the R configuration of compound **2m** with the moderate rigid framework composed of pyrimidine ring and hydroxyphenyl ring, which may be easily to give 5-fluorouracil.

## Figures and Tables

**Table 1:** Inhibitory rates (%) against HL-60.

Compounds	Concentration (mol/L)				
	10^−4^	10^−5^	10^−6^	10^−7^	10^−8^
**2a**	2.0	8.0	9,6	6.9	8.3
**2b**	3.7	5.2	6.2	9.7	2.8
**2c**	33.1	0.1	9.9	9.8	0
**2d**	27.3	0	1.7	0	10.0
**2e-1**	22.4	11.2	7.1	4.2	1.2
**2e-2**	18.5	15.3	3.4	10.5	0
**2f-1**	3.3	10.8	6.6	12.3	0
**2f-2**	31.8	9.2	3.7	6.2	0
**2g**	36.1	10.0	5.7	5.0	2.5
**2h**	55.7	19.6	23.1	2.3	8.6
**2i**	2.8	6.7	1.5	8.3	0.6
**2j**	55.8	12.8	2.7	5.4	5.9
**2k-1**	29.2	0	3.8	8.2	2.4
**2k-2**	51.2	15.5	9.8	12.7	8.9
**2l**	42.4	7.9	5.2	9.9	5.7
**2m**	65.1	0	12.6	13.0	0.3
**2n**	11.4	0	0	0	0
**2o**	22.4	11.2	7.1	4.2	1.2
**5-FU**	57.4	33.5	0	7.0	10.4
**FT-207**	0	0	0	0	0

**Table 2:** Inhibitory rates (%) against BEL-7402.

Compounds	Concentration (mol/L)				
	10^−4^	10^−5^	10^−6^	10^−7^	10^−8^
**2a**	0	0	0	0	0
**2b**	9.0	8.1	0	0	1.8
**2c**	50.0	13.2	5.7	5.2	0
**2d**	13.2	0	0	0	0
**2e-1**	41.2	9.7	8.8	8.2	9.1
**2e-2**	38.4	9.1	8.8	0	5.3
**2f-1**	17.4	10.5	16.6	14.0	4.6
**2f-2**	41.2	9.8	0	0	0
**2g**	36.1	11.1	7.0	7.4	2.4
**2h**	52.6	15.9	2.4	0.7	0
**2i**	14.5	8.7	8.0	4.6	11.7
**2j**	34.0	9.2	4.1	3.5	5.9
**2k-1**	35.9	0	6.9	3.0	0.7
**2k-2**	22.4	11.9	7.1	4.2	1.2
**2l**	36.2	10.2	4.9	0.5	0
**2m**	71.7	68.3	60.4	43.1	24.3
**2n**	8.2	4.5	5.2	0	0
**2o**	9.7	0	8.8	8.2	9.1
**5-FU**	72.6	53.8	35.0	23.8	16.6
**FT-207**	58.0	8.1	0	0	0

## References

[1] Smith N.F., Figg W.D., Sparreboom A. (2004). Recent advances in pharmacogenetic approaches to anticancer drug development. Drug Develop. Res..

[2] Ragnhammar P., Blomgren H.A. (1995). How to optimize the effect of 5-fluorouracil modulated therapy in advanced colorectal cancer. Med. Oncol..

[3] Arias J.L. (2008). Novel strategies to improve the anticancer action of 5-fluorouracil by using drug delivery systems. Molecules.

[4] Nichifor M., Schacht E.H. (1994). Synthesis of peptide derivatives of 5-fluorouracil. Tetrahedron.

[5] Sloan K.B., Wasdo S. (2003). Designing for topical delivery: Prodrugs can make the difference. Med. Res. Rev..

[6] Krishnaiah Y.S.R., Satyanarayana V., Kumar B.D., Karthikeyan R.S. (2002). *In vitro* drug release studies on guar gum-based colon targeted oral drug delivery systems of 5-fluorouracil. Eur. J. Pharm. Sci..

[7] Lee S.M, Lee N.J., Ha C.K., Cho W.J. (2004). Syntheses and biological activities of polymers containing methacryloyl-2-oxy-1,2,3-propanetricarboxylic acid of 5-fluorouracil. J. Appl. Polym. Sci..

[8] Nichifor M, Schacht E.H., Seymour L.W. (1997). Polymeric prodrug of 5-fluorouracil. J. Control. Release.

[9] Yang Y.W., Lee J.S., Kim I., Jung Y.J., Kim Y.M. (2007). Synthesis and properties of N-nicotinoyl-2-(5-fluorouracil-1-yl)-D,L-glycine ester as a prodrug of 5-fluorouracil for rectal administration. Er. J. Pharm. Biopharm..

[10] Elorza B., Elorza M.A., Frutos G., Chantres J.R. (1993). Characterization of 5-fluorouracil loaded liposomes prepared by reverse-phase evaporation or freezing-thawing extrusion methods: Study of drug release. Biochim. Biophys. Acta.

[11] Kratz F., Müller L.A., Ryppa C., Warnecke A. (2008). Prodrug strategies in anticancer chemotherapy. ChemMedChem.

[12] Liu X.J., Chen R.Y., Yang Y.Y. (2002). Synthesis and anticancer activities of novel 5-fluorouracil-1-yl phosphonotripeptides. Chem. J. Chin. Univ..

[13] Yin P., Hu M.L., Hu L.C. (2008). Synthesis, structural characterization and anticarcinogenic activity of a new Gly-Gly dipeptide derivative: Methyl 2-(2-(5-fluoro-2,4-dioxo-3,4-dihydropyrimidin-1(2*H*)-yl)acetamido) acetate. J. Mol. Struct..

[14] Xiong J., Lei X.X., Hu M.L., Yuan J.X., Cai X.Q. (2006). Crystal structure of 2-(5-fluoro-2,4-dioxo-1,2,3,4-tetrahydropyrimidine) glycin, C_8_H_8_FN_3_O_5_. Z. Kristallogr. NCS..

[15] Yin P., Hu M.L., Xiong J., Yuan J.X. (2006). Crystal structure of 2(*R*)-(5-fluorouracil-1-yl)acetyl-2-phenylglycin dimethylformamide solvate, C_14_H_12_FN_3_O_5_·C_3_H_7_NO. Z. Kristallogr. NCS..

[16] Yin P., Hu M.L., Xiong J., Cai X.Q. (2006). Methyl (2S)-2-[2-(5-fluoro-2,4-dioxo-3,4-dihydropy-rimidin-1-yl)acetamido] propionate monohydrate. Acta Crystallogr. E.

[17] Kosynkina L., Wang W., Liang T.C. (1994). A Convenient Synthesis of Chrial Peptide Nucleic Acid (PNA) Monomers. Tetrahedron Lett..

[18] König W., Geiger R. (1970). A new method for synthesis of peptides: activation of the carboxyl group with dicyclohexyl carbodiimide using 1-hydroxybenzotriazoles as additives. Chem. Ber..

[19] Suresh Babu V.V., Gopi H.N. (1998). Rapid and efficient synthesis of peptide fragments containing α- aminoisobutyric acid using fmoc-amino acid chlorides/potassium salt of 1-hydroxybenzotriazole. Tetrahedron Lett..

[20] Han S.Y., Kim Y.A. (2004). Recent development of peptide coupling reagents in organic synthesis. Tetrahedron.

[21] Kuroda M., Mimaki Y., Sashida Y., Hirano T., Oka K., Dobashi A. (1997). Novel cholestane glycosides form the bulbs of ornithogalum saundersiae and their cytostatic activity on leukemia HL-60 and Molt-4 Cells. Tetrahedron.

[22] Skehan P., Storeng R., Scudiero D., Monks A., McMahon J., Vistica D., Warren J.T., Bokesch H., Kenney S., Boyd M.R. (1990). New colorimetric cytotoxicity assay for anticancer-drug screening. J. Nat. Cancer Inst..

[23] Mori M., Hatta H., Nishimoto S.I. (2000). Stereoelectronic effect on one-electron reductive release of 5-fluorouracil from 5-fluoro-1-(2’-oxocycloalkyl)uracil as a new class of radiation-actvivated antitumor prodrugs. J.Org. Chem..

